# Dot size effects of nanocrystalline germanium on charging dynamics of memory devices

**DOI:** 10.1186/1556-276X-8-21

**Published:** 2013-01-10

**Authors:** Ling-Feng Mao

**Affiliations:** 1 Institute of Intelligent Structure and System, School of Urban Rail Transportation, Soochow University, Suzhou, 215006, China

**Keywords:** Quantum size, Nanocrystalline, Tunneling, Memory devices, 85.30.Tv, 85.35.-p, 73.63.-b

## Abstract

The dot size of nanocrystalline germanium (NC Ge) which impacts on the charging dynamics of memory devices has been theoretically investigated. The calculations demonstrate that the charge stored in the NC Ge layer and the charging current at a given oxide voltage depend on the dot size especially on a few nanometers. They have also been found to obey the tendency of initial increase, then saturation, and lastly, decrease with increasing dot size at any given charging time, which is caused by a compromise between the effects of the lowest conduction states and the capacitance of NC Ge layer on the tunneling. The experimental data from literature have also been used to compare and validate the theoretical analysis.

## Background

Memory structures based on Ge nanocrystals (NCs) have received much attention for the next-generation nonvolatile memory devices due to their extended scalability and improved memory performance [1-7]. There are numerous ways of synthesizing Ge NCs. The mean diameter (*d*) of nanocrystalline germanium (NC Ge) using molecular beam epitaxy is uniquely controlled by the nominal thickness (of the deposited amorphous Ge according to the law *d* ≈ *Kt* with *K* ~ 7 using molecular beam epitaxy [1,2]. Comparison of electron and hole charge dynamics in NC Ge flash memories has been discussed in [3].

As we know, the crystal size of semiconductor less than 100 nm can lead to a larger band gap and a change in dielectric constant. In the former work [8,9], the effect of silicon grain size on the performance of thin-film transistors has been studied. To explore NC Ge in a memory device, it is worthy to study how the crystal size of NC Ge on charging dynamics works.

## Methods

### Theory

The energy of the highest valence state (*E*_v_) and the energy of the lowest conduction state (*E*_c_) for spherical NCs of diameter *d* (given in nanometer) are given by the following expression [3]

(1) $$E_{c}\left(d\right)=E_{c}\left(\infty \right)+\frac{11863.7}{d^{2}+2.391d+4.252}\;\left(\mathrm{meV}\right)$$

(2) $$E_{v}\left(d\right)=E_{v}\left(\infty \right)-\frac{15143.8}{d^{2}+6.465d+2.546}\;\left(\mathrm{meV}\right)\text{.}$$

The mean diameter (*d*) of Ge NCs is uniquely controlled by the nominal thickness (*t*) of the deposited amorphous Ge using molecular beam epitaxy according to the law [1,2]

(3) d≈Kt

where *K* ~ 7 uses molecular beam epitaxy. The average density of Ge NCs according to the law [1,2] is

(4) $$D_{\mathrm{NC}}\approx 6\times 10^{-3}/t^{2}\text{.}$$

Note that the Ge NCs have a truncated spherical form and present an aspect ratio (height over diameter) of about 0.8 [1,2]. Thus the filling factor that is the ratio of area of Ge NCs to the total area can be obtained as

(5) $$f=\frac{6\times 10^{-3}}{t^{2}}\times \pi {\left(\frac{d}{2}\right)}^{2}=0.2309\text{.}$$

The self-capacitance of an approximately spherical Ge NC is [6]

(6) $$C_{\mathrm{NC-GE}}\approx 2\pi \varepsilon_{0}\varepsilon_{\mathrm{a-Si}}d\text{,}$$

where *ε*_a-Si_ is the relative dielectric constant of amorphous Si. The capacitance of the amorphous Ge layer is

(7) $$C_{\mathrm{a-GE}}=\varepsilon_{0}\varepsilon_{\mathrm{a-GE}}/t\text{.}$$

Those capacitors are in parallel; thus, the capacitance of the deposited NC Ge layer according to Equations 3, 4, 5, and 6 is

(8) $$C_{\mathrm{Ge}}=\left(0.7691\varepsilon_{\mathrm{a-Ge}}+0.084\pi \varepsilon_{\mathrm{a-Si}}\right)\varepsilon_{0}\times K/d\text{,}$$

where *ε*_a-Ge_ is the relative dielectric constant of amorphous Ge. When Ge NCs in the deposited amorphous Ge layer is charged with one elementary charge by the tunneling electron, causing a voltage buildup *V* = *Q*/*C*_nc-Ge_, hence the amount of energy stored in this layer is

(9) $$E=Q^{2}/\left(2C_{\mathrm{nc-Ge}}\right)\text{.}$$

The total capacitances between gate and substrate are the series capacitances of tunneling oxide, NC Ge layer, and control oxide

(10) $$1/C=1/C_{\mathrm{t-ox}}+1/C_{\mathrm{nc-Ge}}+1/C_{\mathrm{c-ox}}\text{.}$$

When the gate is applied with a positive voltage, the electric field in the tunneling oxide layer in a NC Ge memory with stored charge can be deduced according to the superposition principle of electric fields. Firstly, considering the case that no charge is stored in the NC Ge layer, the oxide field can be obtained as

(11) $$E_{\mathrm{t-ox}1}=V_{\mathrm{ox}}/\left(1+C_{\mathrm{t-ox}}/C_{\mathrm{nc-Ge}}+C_{\mathrm{t-ox}}/C_{\mathrm{c-ox}}\right)/d_{\mathrm{t-ox}}\text{,}$$

where *d*_t-ox_ is the tunneling oxide layer thickness. On the other hand, the dielectric constant of NC Ge can be obtained as $\varepsilon \left(d\right)=1+\left(\varepsilon_{b}-1\right)/\left(1+{\left(\frac{d_{0}}{d}\right)}^{1.1}\right)$[5] (*ε*_b_ is the dielectric constant of bulk germanium). The characteristic radius *d*_0_ for Ge is 3.5 nm. According to the simple superposition formula, the dielectric constant of NC Ge layer is

(12) $$\varepsilon =\mathrm{f\varepsilon}\left(d\right)+\left(1-f\right)\varepsilon_{b}$$

Secondly, the electric field in the tunneling oxide layer in a NC Ge memory with stored charge when the gate is applied with a positive voltage via solution to the Possion’s equation under the boundary conditions can be deduced as

(13) $$E_{\mathrm{t-ox}}=\frac{V_{\mathrm{ox}}+\frac{\sigma }{\varepsilon_{2}}d_{2}\;\left(\frac{\varepsilon_{2}}{\varepsilon_{3}}d_{3}+\frac{1}{2}d_{2}\right)}{\left(d_{1}+\frac{\varepsilon_{1}}{\varepsilon_{3}}d_{3}+\frac{\varepsilon_{1}}{\varepsilon_{2}}d_{2}\right)}\text{,}$$

where *d*_1_, *d*_2_, and *d*_3_ are the thicknesses of the tunneling oxide layer, nc-Ge layer, and control oxide layer, respectively; *ε*_1_, *ε*_2_, and *ε*_3_ are the thicknesses of the tunneling oxide layer, nc-Ge layer, and control oxide layer, respectively; *σ* is the area density of the stored charge. The stored charge density can be calculated using

(14) $$\frac{\mathrm{dQ}}{\mathrm{dt}}=-J_{\mathrm{t-ox}}+J_{g}\text{,}$$

where *J*_t-ox_ and *J*_g_ are the tunneling currents through the tunneling oxide and the gate leakage current, respectively. They have been calculated by using the following equation [10]:

(15) $$J=\int_{0}^{\infty }\frac{qm_{z}^{*}k_{B}T}{2\pi^{2}\hbar^{3}}T\left(E_{z}\right)ln\left(\frac{1+\exp\left(E_{\mathrm{f-L}}-E_{z}\right)}{1+\exp\left(E_{\mathrm{f-R}}-E_{z}-qV_{\mathrm{ox}}\right)}\right)dE_{z}\text{,}$$

where *m*_z_^*^ is the effective electron mass in the silicon along the tunneling direction; *E*_f-L_ and *E*_f-R_ are the Fermi levels of the left contact and the right contact, respectively. The transmission coefficient can be calculated using transfer matrix method. Thus, the tunneling current through the tunneling oxide layer and the gate leakage current can be calculated.

## Results and discussion

In this letter, the effective electron mass 0.5 *m*_0_ of SiO_2_, 0.26 *m*_0_ of silicon, 0.23 *m*_0_ of amorphous Si (a-Si), 0.12 *m*_0_ of NC Ge [11], the relative dielectric constant of SiO_2_, Si, a-Si, and Ge of 3.9, 11.9, 13.5, and 16, respectively have been used in the calculations [12]. The published electron affinities of crystalline silicon, amorphous silicon, SiO_2_, and Ge are 4.05, 3.93, 0.9, and 4.0 eV, respectively [13]. In all calculations except the comparison between theory and experiment, the initial voltage across the total oxide containing NC Ge layer is 10 V, and the tunneling and control oxide thickness are 4 and 25 nm, respectively.

**Figure 1:**
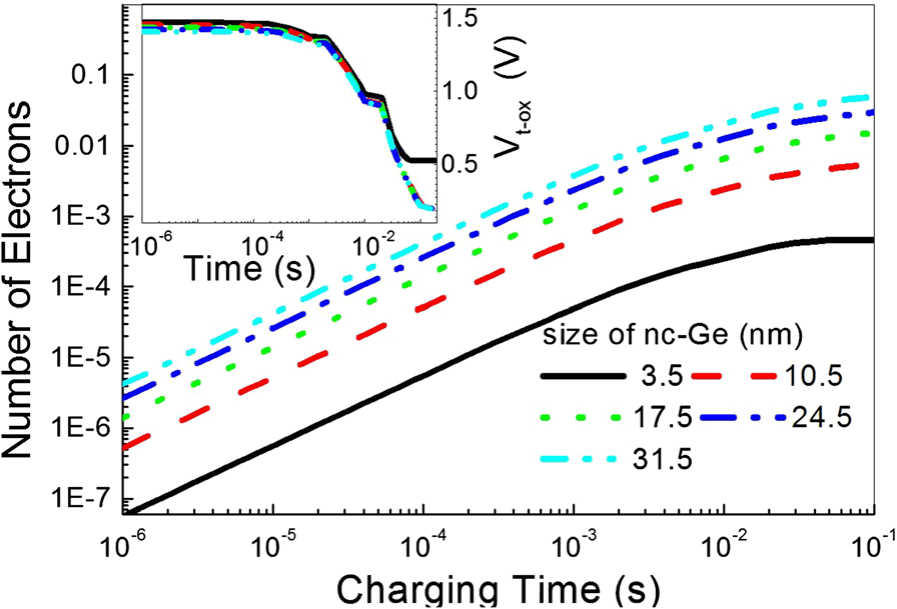
**Average number of electrons per NC Ge dot and the voltage across the tunneling oxide layer.** Average number of electrons per NC Ge dot and the voltage across the tunneling oxide layer as a function of charging time for different sizes.

Figure 1 clearly demonstrates that the average number of electrons per NC Ge dot at the same charging time increases with decreasing dot size. Note that the average density of Ge NCs increases with decreasing dot size according to Equation 4, thus it will need more charging time for the smaller dot size. In addition the voltage across the tunneling oxide layer, which is initially kept constant then slowly decreased and lastly rapidly decreased with charging time, can be concluded from the inset. This is because tunneling electrons captured by NC Ge layer can lead to an inverse static electric field in the tunneling oxide layer and thus, a lower voltage occurs.

**Figure 2:**
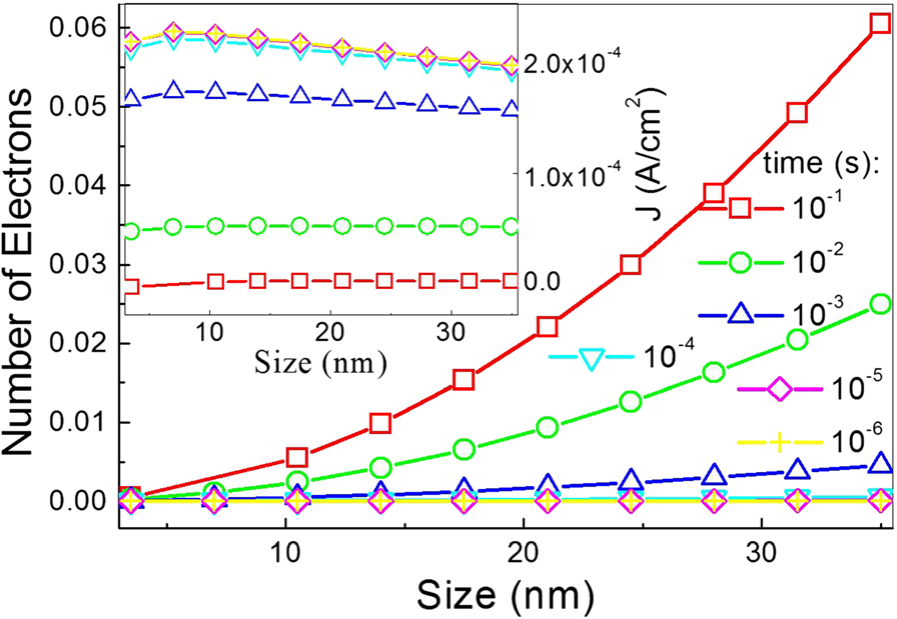
**Average number of electrons per NC Ge dot and charging current.** Average number of electrons per NC Ge dot and charging current as a function of dot size at different charging times.

Figure 2 shows that the average number of electrons per NC Ge dot at any given charging time exponentially increases with the dot size. At the same time, the charging current is found to be initially rapidly increased, then saturated and lastly, slowly decreased with the increasing dot size. It is because the lowest conduction state lowers with increasing dot size according to Equation 1. This denotes that the tunneling probability from the NC Ge layer to the substrates decreases and leads to an increase in the charging current according to Equation 15. On the other hand, the capacitance of NC Ge layer decreases with increasing dot size according to Equation 8 and leads to a larger voltage drop across the NC Ge layer. It implies a lower voltage drop across the tunneling oxide layer and a smaller charging current. The phenomenon about the charging current observed in Figure 2 is a compromise between the effects of the lowest conduction states and the capacitance of NCGe layer on the tunneling.

**Figure 3:**
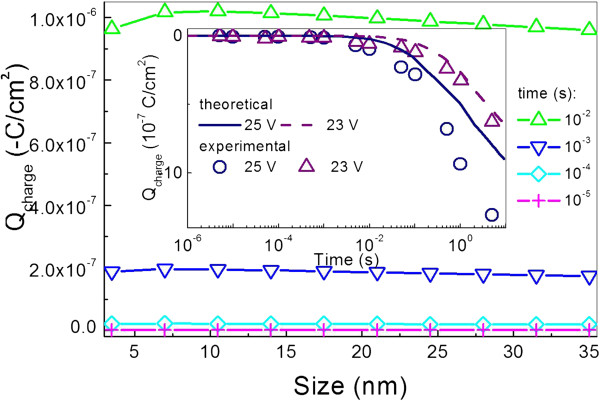
**The stored charge in the NC Ge layer as a function of dot size at different charging times.** Comparison between theoretical and experimental is given as the inset.

Figure 3 depicts how the stored charge in the NC Ge layer changes with dot size at different charging times. One can find that the stored charge in the NC Ge layer initially rapidly increases, then saturates, and lastly, very slowly decreases with increasing dot size at any given charging time. In order to validate the theory, a comparison between the theoretical data using the parameters in [7] and experimental data from the same study [7] is given as the inset figure. The inset figure clearly illustrates that the qualitative theory agrees well with the experiments. The deviance in quantity might origin from the charge captured by the defects in the oxide and NC Ge layer, inappropriate data about effective electron mass for the oxide and NC Ge layer, barrier height between silicon substrate and ultrathin tunneling oxide layer used in the calculation, and overestimation of the capacitance of the NC Ge layer.

## Conclusions

In conclusion, the stored charge and the charging current of NC Ge memory devices with the mean diameter of NC Ge being uniquely controlled by the nominal thickness of the deposition of Ge layer using molecular beam epitaxy are initially increased, then saturated and lastly, decreased with increasing dot size. It is caused by a compromise between the effects of the lowest conduction states and the capacitance of NC Ge layer on the tunneling. Theoretical analysis also demonstrates that the voltage across the tunneling oxide layer is initially kept constant, then slowly decreased and lastly, rapidly decreased with charging time. It is worthy of being noted that NC Ge memory devices may suffer from a small charging current, especially on a few nanometers, due to the change in the lowest conduction states and the capacitance of NC Ge layer.

## Competing interest

The author declares that he has no competing interest.

## Authors’ information

LFM received the Ph.D degree in microelectronics and Solid State Electronics from the Peking University, Beijing, People’s Republic of China in 2001. He is a professor in Soochow University. His research activities include modeling and characterization of quantum effects in MOSFETs and semiconductors, quantum devices, and the fabrication and modeling of integrated optic devices.
